# Secure Vehicular Platoon Management against Sybil Attacks

**DOI:** 10.3390/s22229000

**Published:** 2022-11-21

**Authors:** Danial Ritzuan Junaidi, Maode Ma, Rong Su

**Affiliations:** 1 School of Electrical and Electronic Engineering, Nanyang Technological University, Singapore 639798, Singapore; 2 College of Engineering, Qatar University, Doha P.O. Box 2713, Qatar

**Keywords:** authentication, digital signature, elliptic curve cryptography, key exchange, platoons, Sybil attack

## Abstract

The capacity of highways has been an ever-present constraint in the 21st century, bringing about the issue of safety with greater likelihoods of traffic accidents occurring. Furthermore, recent global oil prices have inflated to record levels. A potential solution lies in vehicular platooning, which has been garnering attention, but its deployment is uncommon due to cyber security concerns. One particular concern is a Sybil attack, by which the admission of fake virtual vehicles into the platoon allows malicious actors to wreak havoc on the platoon itself. In this paper, we propose a secure management scheme for platoons that can protect major events that occur in the platoon operations against Sybil attacks. Both vehicle identity and message exchanged are authenticated by adopting key exchange, digital signature and encryption schemes based on elliptic curve cryptography (ECC). Noteworthy features of the scheme include providing perfect forward secrecy and both group forward and backward secrecy to preserve the privacy of vehicles and platoons. Typical malicious attacks such as replay and man-in-the-middle attacks for example can also be resisted. A formal evaluation of the security functionality of the scheme by the Canetti–Krawczyk (CK) adversary and the random oracle model as well as a brief computational verification by CryptoVerif were conducted. Finally, the performance of the proposed scheme was evaluated to show its time and space efficiency.

## 1. Introduction

The spontaneous formation of a network of mobile devices interconnected wirelessly has introduced the concept of mobile ad hoc networks (MANETs). In recent years, the iteration of MANETs utilizing vehicles as mobile devices has evolved the research field to what it is known as today: vehicular ad hoc networks (VANETs). Throughout their evolution, VANETs have seen much progress in terms of different applications. One notable application that has been gaining much interest is vehicle platooning. Platooning, in brief, involves having a manually driven vehicle, known as a platoon leader, spearheading a convoy of semi-automated vehicles in a single lane on the road. These vehicles can be referred to as platoon members, and among this train of vehicles, they are all spaced closely and uniformly apart from each other [1].

The benefits of platooning include decreasing the headway time (gap) between platoon members, thereby providing better traffic management. A platoon can also help to increase the capacity on highways as vehicles in the platoon take up less space than when vehicles are independently and individually controlled [2]. Other known benefits of platooning include cutting down on fuel consumption due to the reduced aerodynamic drag from the slipstream effect of travelling in a close convoy [3], increased driving comfort and the removal of human errors in traffic accidents since trailing vehicles can be semi-autonomously driven. The smoother cruise also reduces engine wear [4].

Due to the automated nature of platooning, vehicles need to periodically broadcast messages containing crucial information such as vehicle identity, position and speed [2]. Hence, vehicle-to-vehicle (V2V) and vehicle-to-infrastructure (V2I) communications are needed to facilitate the broadcast of such messages among the vehicles as well as to base stations. These base stations are situated along the sides of roads and are known as roadside units (RSUs). Interactions with RSUs and other platoon members provide crucial information such as knowing whom to interact with and how to behave while inside the platoon, thereby allowing the vehicles to move in tandem smoothly [5]. However, if this network and its supporting actors are compromised, it could potentially cause the platoon to be unsafe.

Such a scenario could occur if the communication channel of the network is disrupted. For instance, vehicle nodes could experience a loss of signal for technical reasons when multiple simultaneous transmissions among vehicles interfere with one another. Vehicles would share the same channel and transmit at the same time, causing transmission packets to collide and subsequently be dropped. Researchers such as in [6] have addressed this issue in the platoon context by devising a channel allocation algorithm based on the orientation of vehicles in a platoon. Since platoon vehicles are ordered in a single file and separated uniformly from one another, vehicles can be allocated specific channels depending on their distances from the platoon leader. In short, only platoon vehicles that are out of the interference range can use the same channel. Their real-world experiment using Android-based mobile devices showed that their scheme outperformed typical platooning systems with regards to packet drop rates and delays.

On the other hand, an alternative hazardous scenario could arise whereby nodes that are intentionally malicious worm their way into the network to influence the behaviors of the vehicles in the platoon. One case where these malicious nodes can inhibit the operation of platoons is through a Sybil attack. The definition of such an attack is when a malicious actor forges fake virtual vehicles via pseudonyms [1] so that it can mask itself as multiple simultaneous vehicles that seem to be physically present on the roads [1,2,7,8,9,10,11]. These illegitimate vehicles, known as Sybil vehicles, can be preloaded with any number of false credentials that are not of the original malicious node [7]. They can also steal the credentials of legitimate vehicles and use the stolen information to impersonate them. If these Sybil vehicles are admitted into platoons, the malicious node can send false messages to the other platoon members through these virtual vehicles. These false messages are shared within the platoon to fabricate fake traffic scenarios. The platoon and its members would then have to consider the given traffic scenario and act accordingly, altering the platoon’s original intentions and affecting its overall performance as a result. Furthermore, within the platoon itself, the sheer presence of these Sybil vehicles causes undesirable gaps to appear between the platoon members as they have to accommodate for the “physical presence” of these virtual Sybil vehicles. In turn, it directly impedes the platooning benefits such as fuel economy and road capacity [8]. Thus, it is imperative that only honest vehicles be authenticated and admitted into platoons.

### Motivation and Contributions

We have reviewed existing research work utilizing technologies of our interest. We then focused on cryptographic-based works and discovered that they exhibited incomplete authentication of vehicle identity and messages. Those that achieve complete authentication only do so at high computational costs.

To overcome the shortcomings of these existing solutions, we designed a hybrid security scheme that authenticated both vehicle identity and messages exchanged into a platoon management scheme to prevent threats by Sybil vehicles. The proposed solution is called secure platoon management against Sybil attacks (SPMSA) and employs the ECC to provide a secure yet lightweight solution against the Sybil attacks hampering platoon operations. Other typical attacks that SPMSA resists include replay, man-in-the-middle and distributed denial-of-service attacks. Lastly, to preserve the privacy of the vehicles and the platoon, our scheme offers perfect forward secrecy as well as group forward and backward secrecy.

The remainder of the paper is as follows: Section 2 discusses existing research work related to platoon management against Sybil attacks. Section 3 discloses the system model and preliminaries. Section 4 describes the SPMSA scheme in detail. The security and performance of SPMSA are evaluated in Section 5 and Section 6, respectively. Section 7 concludes the paper.

## 2. Related Works

In this section, we provide a holistic overview of existing research work that has addressed the problem of Sybil attacks on vehicular platoons. Although various technologies have been used to address this research problem, we only focus on three main technologies: blockchain, machine learning and cryptography. However, investigators found that there is a scarcity of work that explicitly addressed securing against Sybil attacks in the context of vehicle platoons [2]. To the best of our knowledge, a general approach was taken by most researchers, who addressed the problem of Sybil attacks in VANETs instead. Nevertheless, we review those works that we believe can be adapted for platooning.

### 2.1. Blockchain

Bochem et al. [12] proposed a fully decentralized blockchain that monetarily disincentivizes the creation of Sybil identities in MANETs. It works with any proof-of-work (PoW)-based blockchains (e.g., Ethereum) and binds vehicular node identities to cryptographic public–private key pairs known as blockchain wallet addresses. Whenever a vehicle wants to join the network, an identity proof is created for it. In this process, the vehicle sends a cryptocurrency deposit to the deposit wallet, where this transaction is subsequently mined into a block in the blockchain. Through the mined block, an identity proof is created for the joining vehicle that contains the details of the transaction and the wallet address (i.e., vehicle’s identity in the form of its public key) responsible for it. Before the vehicle nodes can start to communicate, they exchange their uniquely generated identity proofs as part of a two-way handshake to verify their peers’ identities and prevent Sybil nodes from entering the network for free. The vehicle it exchanges its identity proof with (i.e., identifier) is selected at random to prevent a scenario where a malicious vehicle colludes with one of its Sybil vehicles to disingenuously validate itself. The scheme was initially used for the offline verification of identity proofs, but Bochem and Leiding [13] later adapted the scheme for Internet-of-Things (IoT) environments as well and included the functionality of revoking false identities: If the identity proof of a vehicle is rejected, the identity is revoked. The identifier deems the vehicle a possible Sybil vehicle, and no further communication takes place between the vehicles. Moreover, since generating new identities is expensive and there are likely to be a considerable number of honest nodes in the network, it is infeasible for a malicious entity to perform attacks utilizing Sybil vehicles. However, disregarding the costs for malicious users, the costs to honest users in this scheme could be cause for concern. As the scheme leverages PoW, the costs of transactions are higher since more blocks are mined, and hence, more energy is consumed. As we stated, the cost of a revocation transaction increased by more than tenfold in just one year.

On the other hand, Liu et al. [14] proposed a dual cyber-physical blockchain instead for building a secure and trusted communication for connected vehicle (CV) applications. Their approach involved using the proof-of-stake (PoS) as its consensus mechanism and adopting sharding to partition vehicles into regions and decrease computation, communication, and storage costs. Two blockchains were used in their scheme: the trust points blockchain and the proof-of-travel blockchain. The former quickly identifies and records malicious misbehavior and can be regarded as a misbehavior detector. Intuitively, telling the truth and being acknowledged by most neighbor vehicles earn trust points, while telling a lie loses trust points. Meanwhile, the second blockchain records each vehicle’s historical contribution to the CV community and can be regarded as a reputation management system. This contribution comes in the form of a vehicle’s travel activities. The more traffic information a vehicle shares with other vehicles around it, the greater its contribution to the CV community, and the more proof-of-travel credits it receives. The outputs of the two blockchains are then added up to form the stake of a vehicle. The higher the vehicle stake, the more trustworthy it is within the network. Hence, Sybil vehicles in the network are detected if they have a small stake from the two blockchains. However, breaking the area into multiple regions does intuitively mean more transfers of records between blockchains in different regions when a vehicle travels frequently between regions. This could accumulate considerable latency as more blockchain mining should occur.

Didouh et al. [15] proposed a novel cyber-physical blockchain architecture to prevent position spoofing attackers such as Sybil nodes from becoming validated nodes for the highway ETC application. The scheme is a witness-based approach using proof-of-location (PoL) as its consensus mechanism. Smart contracts were used as an authentication method to determine the legitimacy of a node. A consortium blockchain was maintained by the RSUs in the network, granting these RSUs access and authority over the network. Thus, only RSUs can mine new blocks and permit nodes into the blockchain. When a vehicle (prover) enters the network, RSUs collect the prover’s PoLs provided by other vehicles (witnesses) and calculate its overall score. Since the witnesses are likely to be honest, the prover’s PoL should prove that the prover is in the position where it claims to be. If the prover’s PoL score is below a specified threshold, it is determined to be a Sybil vehicle and denied entry into the network. To ensure that the PoL scores are transparent for any node to view and verify the information without the need for an external party, smart contracts were published in the blockchain as their nonrepudiation nature guarantees that no vehicle can deny the authenticity of its information on the blockchain.

### 2.2. Machine Learning

A form of machine learning is the support vector machine (SVM), which Gu et al. [16] used to detect Sybil attacks in VANETs. The SVM was used to classify the driving patterns of vehicles such that Sybil vehicles could be distinguished from legitimate ones. The flexible nature and good generalization performance of the SVM allow it to reduce overfitting of the data and solve various problems without requiring major tuning. This makes SVM a suitable learning algorithm for the dynamic environment of VANETs. The driving patterns of vehicles are thus defined as aggregations of the quantifiable vehicle data, which include time, location, velocity, acceleration and acceleration variation. Crucially, the scheme works on the hypothesis that when traffic is dense, vehicles drive in a similar manner, and hence, any obvious outliers with relatively high variance in their driving patterns can be detected as Sybil vehicles.

Similarly, the authors in [17] used the mobility information of vehicles sent to the RSUs to form the input matrix that represents the driving patterns of the vehicles. Rather than use an SVM, their scheme used extreme learning machine (ELM) to determine the similarity of the mobility patterns and detect the Sybil nodes. ELM was used over traditional artificial neural networks due to its greater advantage with regards to stopping criteria, learning rate and minimum local and over-tuning, contributing to a faster and lower complexity feed-forward learning algorithm. In both schemes [16,17], however, there are doubts as to how effective they are when traffic density is low because then there is greater variation in the benign vehicles’ driving patterns.

The concept of radio frequency (RF) fingerprinting was explored by [18], who inserted signatures into transmitted I (in-phase) and Q (quadrature) samples so that a transmitter could be passively identified as a friendly or authorized party. This was carried out by the detection of these signatures through a deep convolutional neural network (CNN) at the physical layer. Reus-Muns et al. [19] adapted this concept for use in 5G and open radio access networks (open RANs) to identify trustworthy base stations instead. Doing so could prevent Sybil attacks whereby base stations attempt to spoof as other base stations. Using real-world datasets, they were able to demonstrate an accuracy of 99.86% irrespective of the training and testing time gap. This outlines the potential benefit of building trust for future open RAN networks and then for use in platoon networks to identify spoofing-based attacks such as Sybil attacks. Similarly, Comert et al. [20] used deep learning-based RF fingerprinting methods to identify the malicious transmitters in cyber-physical systems. However, the authors found that using real-world datasets that considered all environmental scenarios was impractical. Hence, they used unobserved data instead and obtained a peak accuracy of 87.94%, a modest drop from the results obtained by [19].

### 2.3. Cryptography

The work in [8] is one such cryptography-based work that has explicitly addressed securing platoons against Sybil attacks. The authors did so by integrating a hybrid key management with a witness-based mechanism as a defense protocol. Evaluations performed using OMNeT++, SUMO and Veins framework showed that Sybil attacks could be significantly deterred with minimal overhead effect on network throughput and delay. The minimal overhead is realized by bootstrapping the credentials of the public key infrastructure to establish pairwise symmetric keys, making the proposed protocol lightweight as a result. The identities of the vehicles are encrypted during transmission and decrypted upon reception with this symmetric key, ensuring the safe transfer of vehicle identities from one vehicle to another. A vehicle in the network is then able to verify whether a neighboring vehicle is a Sybil node or not by inspecting its witness table. The witness table holds the information of the other vehicles (witnesses) it passed along the way to reach the verifier node, i.e., the route to the verifier vehicle. If there are missing witnesses, the verifier deems the route invalid, and the node is detected as a Sybil vehicle. Hence, by symmetric key cryptography and the witness-based algorithm, vehicle identities are authenticated. However, the scheme is unable to verify the authenticity of messages exchanged among the vehicles, which brings about the possibility of supposed trustworthy vehicles sending false messages.

Authors in [9] proposed privacy-aware Sybil attack detection (PASAD) that aimed to detect Sybil attacks in both V2V and V2I communications while preserving vehicle privacy. The proposed scheme adopted the Boneh–Shacham (BS) short group signature and safe physical authentication to set up a secure and privacy-aware communication channel for the vehicles. The PASAD scheme is able to detect Sybil attacks on two levels. The first level detects Sybil attacks from an outsider attacker by checking unique registrations of vehicles for any double registrations. This level of detection utilizes the safe physical authentication in trusted RSUs (TRSU) and the BS group signature in RSUs. Meanwhile, the second level prevents insider Sybil attacks by checking for the retransmission of warning messages in V2V communication and double-registrations of vehicles. At this level, the BS group signature is used to ensure the uniqueness of warning messages and the vehicles that sent them. In short, PASAD is able to prevent both outsider and insider attacks in a decentralized manner, even when vehicles are out of range of the RSUs. Thus, the scheme can achieve authentication by verifying both a vehicle’s physical presence and the short group signatures attached to the broadcasted warning messages. Although additional overhead is not imposed by the scheme, its increase in computational delay when there are many invalid signatures is a cause for concern when VANETs have to be time efficient.

An alternative approach to detecting Sybil vehicles is seen in [10], who used timestamps in conjunction with a hybrid public key infrastructure. Specifically, a chain timestamp was utilized to provide secure communication in the public channel. The proposed scheme concatenated an encrypted message with a timestamp before it was transmitted to the receiver. Another timestamp was generated upon the receipt of the message, and each timestamp was subsequently recorded by the RSU. Under the chain timestamp concept, if the RSU records consecutive timestamps that are the same, then a Sybil attack has been detected and the session concludes. Otherwise, the message will be sent to the receiver as normal. However, it is not shown how the identities of the vehicles are secured and hence validated. Although a public key architecture is in place to ensure that a trusted authority is in control of the private–public key pairs of the vehicles, the message exchange between a sender and receiver only requires the receiver’s set of private and public keys. As it is a one-way communication system, the receiver is unaware of the identity of the sender, who could very well be a malicious node. In such a scenario, the message received might also not be safe. Thus, expanding the proposed scheme to authenticate the identity of the sender and the messages received could help to provide greater security.

One work that adopted message authentication is [11], where elliptic curve cryptography (ECC)-based digital signatures were attached to messages to verify their origin and authenticity, thereby strengthening the vehicle’s privacy against Sybil attacks. The elliptic curve digital signature algorithm (ECDSA) is a secure hash algorithm that generates and verifies these signatures. When a vehicle sends a message, it signs the message using its private key. The receiver is then able to verify the digital signature attached to the message by using the sender’s public key. If the verification fails, the signature is rejected, and the message is suspected of coming from a Sybil vehicle instead. Moreover, even if an attacker were to steal this signature and use it as its own when communicating with other vehicles, the verification process would still fail because the signature had been generated using the private key and message of the original vehicle. The minimal delay in signature generation and verification implies that the efficiency of the proposed scheme is dependent on the traffic and message size instead. The proposed ECDSA can provide greater security and safety for the vehicles during message transmission while being more time and memory space efficient than other key cryptographic algorithms. In a future plan, one suggestion was to extend the proposed scheme by assigning and registering unique identification numbers (IDs) for vehicles to authenticate their identities. Although this would ensure greater message security, it would also inherently increase the processing time.

## 3. System Model and Preliminaries

### 3.1. System Model

**Figure 1:**
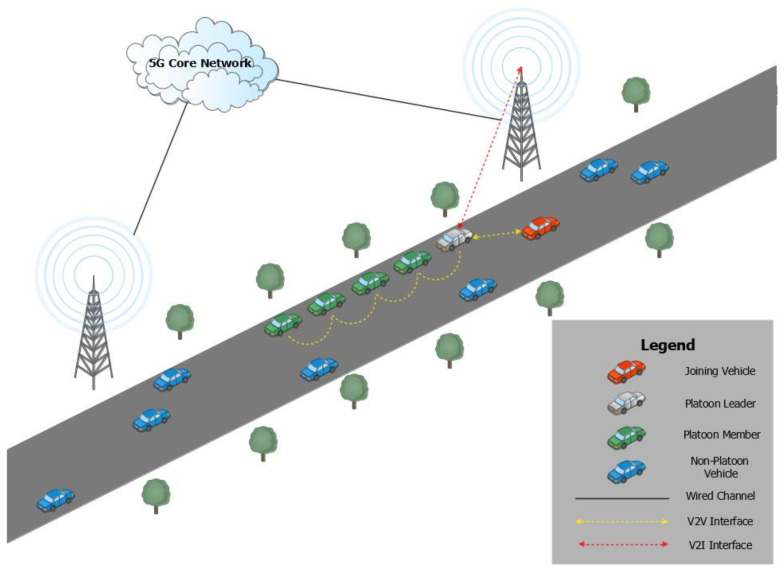
System architecture.

A typical highway scenario is considered wherein only vehicles equipped with the appropriate on-board unit (OBU) can communicate wirelessly with other similarly equipped vehicles and trusted RSUs within the VANET. Hence, these OBUs facilitate the vehicles’ communications. The fifth-generation cellular technology (5G), which is protected by the security scheme specified as part of the Third Generation Partnership Project (3GPP) standard, is used as the means for the V2I communication between the vehicles and the RSU. Meanwhile, V2V communication that allowed vehicles to talk to one another utilized dedicated short-range communication (DSRC). Each vehicle’s OBU was equipped with two separate interfaces to allow for parallel V2I and V2V communications via 5G and DSRC, respectively. A 5G core network database that was used to securely store the vehicles’ confidential data has a wired connection to each RSU. At least one platoon led by a platoon leader is assumed to be present in the VANET at all times of the platoon operation. The role of the platoon leader is assumed to remain with the same vehicle while the proposed scheme is being run. In addition, no platoon members can communicate with any entities outside of the platoon except for the platoon leader. A simple illustration of the architecture of the system under the study can be found in Figure 1. In this paper, we focus on the management of a platoon when a vehicle intends to join the platoon to the moment it eventually leaves the same platoon.

### 3.2. Threat Model

The threat model we consider is the Canetti–Krawczyk (CK) adversary model [21,22,23], which is defined as follows:

1. Participants: Let JV be the vehicle attempting to join a platoon, PL be the leader of the specific platoon, RSU be a trusted RSU and P be any of the participants. All the participants are considered oracles.
2. Partners: If two oracles. e.g., JV and PL, share the same session key, then they are known as partners.
3. Adversary: 𝒜 represents a Sybil vehicle adversary running in polynomial bounded time that can attack by eavesdropping, modifying, injecting messages, etc.
4. Queries: Various actions 𝒜 can make are defined in the following queries:
  - Send(P,m): 𝒜 modifies and sends the message m to P, then receiving a response from P.
  - Execute(JV,PL): 𝒜 passively eavesdrops on the communication between JV and PL, returning a copy of the information exchanged between the two participants.
  - Corrupt(P): 𝒜 obtains a long-term private key of P.
  - ESReveal(P): 𝒜 obtains the ephemeral private key of P.
  - SKReveal(P): 𝒜 obtains the session key of P.
  - Expire(P): 𝒜 deletes a completed session key of P.
  - Hash(m): 𝒜 obtains random hash r due to the hashing of message m, i.e., Hash(m)=r. Any subsequent Hash(m) of the same m produces the same r.
  - Test(P): Used to test a session key’s semantic security. An unbiased coin c is flipped. If c=1, session key of P is sent to 𝒜. Otherwise, a random value is sent to 𝒜.
5. Semantic Security: A semantically secure system is one in which within a reasonable amount of time, it is infeasible for 𝒜 to obtain significant information about a plaintext message given only its ciphertext. Given that 𝒜’s objective is to predict the result of a test query correctly, let Pr[S] denote the probability that 𝒜 succeeds in its prediction. Subsequently, the advantage of 𝒜 in breaking the semantic security is generally defined as Adv(A)=|2Pr[S]−1|. Hence, if Adv(A)≤ε is satisfied for any sufficiently small value ε>0, the scheme is safe by the CK adversary model.

### 3.3. Elliptic Curve Cryptography

ECC is a type of public key cryptography that offers security equivalent to more widely used cryptosystems such as the Rivest–Shamir–Adleman (RSA) algorithm while requiring fewer bits for computation [11,22]. Hence, the ECC is ideal for systems that are limited in terms of storage, bandwidth and power [11].

An elliptic curve in its simplest form satisfies the equation $y^{2}=x^{3}+Ax+B$, where $A,B\in F_{P}$ are constants with $4A^{3}+27B^{2}\neq 0\;mod\;p$ and p ≥5 is a prime number. Hence, the constants A and B control the elliptic curve that is produced. The set of (x,y) 2-tuple that satisfies the elliptic curve equation lies on the curve itself and is referred to as $E(F_{P})$. One pair that fundamentally exists for an elliptic curve used in a system deploying ECC is the generator point G, i.e., G is a point on the curve satisfying the equation and has the coordinates of (x,y). Let d represent the private key, which is chosen randomly in the interval [1,n−1], where n is the order of G greater than $2^{Bit\;Size}$. This scalar multiplication of d and G would then produce the public key D i.e., D=dG. It forms the basis of the ECC, which works on the elliptic curve private–public key pair (d,D). Lastly, the security of the ECC is guaranteed if the following computations hold [22]:

1. Elliptic curve discrete logarithm problem (ECDLP): Given two points $G\in E(F_{P})$ and $aG\in E(F_{P})$, it is computationally hard for a polynomial time bound algorithm to compute $a\in F_{P}$.
2. Elliptic curve computational Diffie-Hellman (ECCDH) problem: Given three points $G\in E(F_{P}),\;aG\in E(F_{P})$ and $bG\in E(F_{P})$, it is computationally hard for a polynomial time-bound algorithm to calculate abG where $a,b\in F_{P}$ are unknown parameters.
3. Elliptic curve decisional Diffie–Hellman (ECDDH) problem: Given four points G, A=aG, B=bG and C=cG in $E(F_{P})$, where a, b and c are unknown parameters and $a,b\in F_{P}$, it is difficult to determine if C=abG.

## 4. Proposed SPMSA 

We proposed the SPMSA with the main motivation of establishing a platoon management scheme that would be secure against Sybil attacks. As mentioned, Sybil attacks against platoons are defined as instances where fake virtual vehicles use forged identities to admit themselves legitimately into platoons so as to disrupt the platoons’ operation. The proposed scheme was designed with the intention of addressing most of the drawbacks of current cryptographic implementations discussed in Section 2.3. These drawbacks include incomplete identity and message authentication as well as high computation costs.

As it concerns platoon management, the proposed scheme operated over three main events: platoon entry, platoon communication and platoon exit. In short, a vehicle’s entire journey from joining the platoon to when it eventually leaves the platoon was covered.

### 4.1. Platoon Entry Event

Platoon entry is defined as when a vehicle is attempting to join a platoon by interacting with its leader who has the authorization of admitting vehicles into its platoon. The major security issue in this event is ascertaining the identity of the joining vehicle. The legitimacy of this identity should be ensured by verifying that it truly is a registered vehicle. Another security issue is verifying that the messages exchanged in the platoon were not altered. Messages could be intercepted, altered and/or repeated by the Sybil vehicles to falsely admit them into the platoon or deny entry to legitimate vehicles. Hence, the hybrid authentication of both identity and message for this event is required and subsequently provided by the SPMSA.

This event is further broken into four individual phases: initialization, identity authentication, message authentication and platoon key update.

#### 4.1.1. Initialization Phase

In the initialization phase, vehicles equipped with OBUs in the VANET first register their unique vehicle IDs to the 5G core network database through an RSU to obtain the common generator point G used throughout the network. The vehicles also obtain their respective unique long-term private–public key pair (d,D) in return, which is used as a pseudonym to preserve identity privacy [1]. Since a malicious node holds multiple identities when carrying out a Sybil attack, each vehicle is only allowed one identity and as such, can only possess one key pair (d,D) at one time. The initial platoon key qG is generated so that all platoon members can use it to communicate with each other. Thus, this platoon key is only shared among the platoon members. Timestamps were added to each message throughout the operation of the platoon to ensure the freshness of messages, where T is the timestamp when a message is sent, while $T^{\prime }$ is the timestamp when the same message is received. Whenever a message is intercepted and relayed to the intended recipient, additional time is taken up for the message transmission. Should the time delta between reception and transmission exceed a specified threshold, that is, the estimated time taken for the message to be in transit, i.e., if $T^{\prime }-T>\sigma$, the message might have been intercepted, so the message and the associated session are discarded. Note that the threshold adjusts independently for each message transmission to accommodate any additional computational operations that could take place before a message is sent out as well as after a message is received. Finally, it is assumed that the ECDLP, ECCDH and ECDDH problems are hard to solve. The initialization phase happens before the start of platoon operation.

#### 4.1.2. Identity Authentication Phase

**Figure 2:**
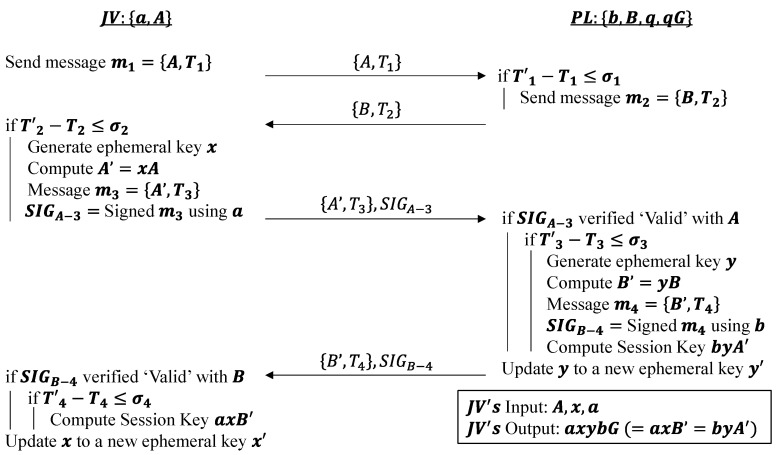
Identity Authentication Phase of the SPMSA.

The identity authentication phase is visualized in Figure 2, where a Sybil vehicle is detected whenever an if’ statement is not fulfilled. It is important to note that a Sybil vehicle is detected in this manner throughout the entire operation of the platoon and not just in this specific phase. The main objective of this phase is for the joining vehicle and platoon leader to generate and agree upon a secret key that only they will share. This secret key is commonly referred to as a session key and is used to encrypt and decrypt messages so that unintended vehicles cannot decipher and read them. It can be seen as an attempt to prevent external attacks, namely Sybil vehicles spoofing as authentic identities.

The ECDSA is used to secure the message. It is an elliptic curve variant of the DSA and boasts a shorter key length than the RSA. The ECDSA allows a sender to sign a message with its own private key and attach the generated digital signature to the message. By verifying the attached signature with the sender’s public key, the recipient can check whether the signature and hence the message it is attached to are authentic and came from the sender instead of an adversary. The ECDSA works as follows [11]:

**Key Generation:**

1. Elliptic Curve Parameters: $A,\;B\in F_{P}$: Domain Parameters; $G\in E(F_{P})$: Generator Point; n: Order of G greater than $2^{256}$; d∈[1, n−1]: Randomly selected Private Key of Sender; D=d·G: Generated Public Key of Sender

**Signature Generation [Input: message** m **,** d **]:**

2. $t\cdot G=\left(A_{1},B_{1}\right)$: A point on elliptic curve of randomly selected number t∈[1, n−1]
3. $r=A_{1}\;mod\;n$: Go back to Step 1 if r=0
4. $s=(t^{-1}\left(SHA\left(m\right)+d*r\right)\;mod\;n$: Go back to Step 1 if s=0, where SHA is the hash function
3. (r,s): Output ECDSA Signature

**Signature Verification [Input: message** m **,** (r,s) **,** D **]:**

6. r,s∉[1,n−1]: Signature is invalid if this condition occurs
7. $w=s^{-1}\left(mod\;n\right)$: Compute w
8. $u_{1}=[\left(SHA\left(m\right)\cdot w\;mod\;n\right]$: Compute $u_{1}$
9. $u_{2}=\left(r\cdot w\right)\;mod\;n$: Compute $u_{2}$
10. $V=\left(u_{1}\cdot G+u_{2}\cdot D\right)\;mod\;n$: Signature is valid if V=r

As a result of the initialization phase, the joining vehicle JV and platoon leader PL own the private–public key pairs (a,A) and (b,B), respectively. To realize this exchange, the existing elliptic curve Diffie–Hellman (ECDH) key exchange protocol is modified, which itself is an ECC-based variant of the original Diffie–Hellman protocol [21]. The identity authentication phase starts with the standard ECDH, where JV and PL exchange their public keys A and B so that they can each compute the same session key thereafter. Upon receiving A, PL can then compute bA=baG. Similarly, after receiving B, JV computes aB=abG, which is equivalent, and thus, a common key is set up between the two vehicles.

However, to secure this secret key further, another round of key exchange between the two vehicles occurs. In this round, both vehicles first generate a random temporary private key known as an ephemeral private key, which expires and is updated to a new random value once the identity authentication phase ends. A corresponding ephemeral public key is then generated such that JV, for example, would have an ephemeral private–public key pair $\left(x,A^{\prime }\;\right)=\left(x,xA\right)$ where x∈[1,n−1]. JV would send to PL its ephemeral public key $A^{\prime }=xA$ with an ECDSA signature $SIG_{A-3}$ attached to it that has been signed using its initial private key a. Upon reception, PL first verifies the signature $SIG_{A-3}$ and then the timestamp $T_{3}$. If either fails to be validated, the message and session are discarded as a potential Sybil vehicle has been detected. Otherwise, PL carries out similar actions as JV and sends back its own signed ephemeral public key as acknowledgment. If all the timestamps and signatures are valid, a session key $axB^{\prime }=byA^{\prime }=axybG$ can be derived and secretly agreed upon on both sides. Finally, the ephemeral private keys of both vehicles are refreshed.

#### 4.1.3. Message Authentication Phase

**Figure 3:**
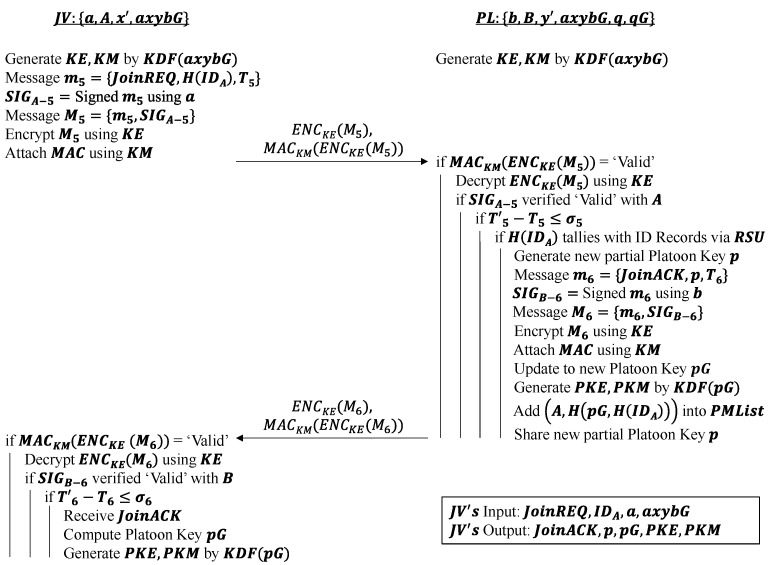
Message Authentication Phase of the SPMSA Scheme.

Figure 3 demonstrates the procedure of the message authentication phase. The main purpose of this phase is to hand the platoon key over to the joining vehicle in a secure manner. Hence, to establish a secure handover of the platoon key, message authentication is used to ensure the data integrity, origin and authenticity of the messages being transmitted. In contrast to identity authentication, message authentication can prevent internal attacks, particularly, which is an attack by a Sybil vehicle that has been authenticated as a legal user within the VANET. A modified version of the elliptic curve variant of integrated encryption scheme (ECIES) is used to provide semantic security in this phase. Briefly, the standard ECIES encrypts a plaintext message and attaches a message authentication code (MAC) to this encrypted message [24]. The MAC is also known as a keyed hash function as it takes as input a secret key (i.e., MAC key) and the encrypted message to produce a hash i.e., MAC as the output. The MAC guarantees the message’s integrity and authenticity because only an actor who has the knowledge of the secret key can generate the MAC [25]. Thus, the receiver can check the MAC for authenticity of the message. Since the receiver shares the same secret key with the sender, if the MAC is deemed invalid by the receiver, then the message might have been tampered and is not safe to be decrypted. The modified ECIES works as follows:

**Encryption of message** m **[Input:** axybG **]:**

1. KDF(axybG)=KE||KM: A Key Derivation Function (KDF) is used to derive Symmetric Encryption Key KE and MAC Key KM from the shared Session Key *axybG*
2. ENC(KE;m)=c: Encrypt message m using Symmetric Encryption Key KE
3. $MAC\left(KM;c\right)=d_{A}$: Generate the MAC tag $d_{A}$ of encrypted message c using MAC Key KM
4. $c||d_{A}$: Ciphertext output of joining vehicle JV

**Decryption of ciphertext** $c||d_{A}$ **[Input:** $c||d_{A}$ **,** axybG **]:**

5. KDF(axybG)=KE||KM: Symmetric Encryption Key KE and MAC Key KM is derived by PL in the same manner as JV did
6. $MAC\left(KM;c\right)=d_{B}$: MAC is Valid’ and encrypted message c has not been tampered with in transit if $d_{B}=d_{A}$
7. $ENC^{-1}\left(KE;c\right)=m$: Decrypt c using Symmetric Encryption Key KM to obtain message m

This phase is initiated by JV sending PL a request to join PL’s platoon with a hash of its ID, and the accompanying ECDSA signature $SIG_{A-5}$ belonging to JV. The session key axybG computed at the end of the identity authentication phase is used as the input to the KDF to derive the symmetric encryption key KE and MAC key KM. The benefit of generating the two keys from the KDF is that key diversification can be achieved. The session key is essentially hashed because a master key (i.e., session key) is being separated into two children keys (i.e., KE and KM). Thus, even if an attacker wants to obtain one of the derived keys, it is unable to reverse engineer the stolen key to get the entire session key or the other derived keys. Thus, JV uses KE to encrypt the concatenated message $M_{5}$, and KM to generate and attach a MAC to the encrypted form of $M_{5}$ before sending them over to PL. It ensures that any tamper attempt on the encrypted message (i.e., joining request, ID, timestamp, and signature) can be checked and detected by the PL through this MAC upon message reception.

Only if the following conditions occur in order is a new partial platoon key *p* generated and JV’s identity added into the platoon members list PMList as a 2-tuple $\left(A,H\left(pG,H\left(ID_{A}\right)\right)\right)$:

1. MAC from JV is valid
2. JV’s digital signature $SIG_{A-5}$ is valid
3. Timestamp delta is within threshold range $\sigma_{5}$
4. Hashed $ID_{A}$ tallies with the ID records in the 5G core network database after the PL sends a database check query through the RSU

If the above conditions are satisfied, a new partial platoon key p is generated and sent to JV using the same message composition procedures conducted by JV at the start of the message authentication phase. It allows JV to compute the updated platoon key pG, thereby authenticating it as an official platoon member PM that can communicate with others from now on. At the same time, PL shares this new partial platoon key p with other current PMs, who will update their platoon key according to the new key pG to preserve the privacy of the previous platoon key qG from the new platoon member, JV. This can ensure group backward secrecy on the old platoon key qG achieved.

Platoon-wide symmetric encryption and MAC key are then generated by a KDF of the common platoon key pG upon JV entering the platoon, and these keys are referred to as PKE and PKM, respectively. Consequently, all authenticated platoon members PMs share the same set of PKE and PKM keys. As can be seen, the use of digital signatures and MACs ensures the origin, data integrity and authenticity of messages exchanged in this phase.

#### 4.1.4. Platoon Key Update Phase (Entry)

This phase occurs whenever a vehicle is authenticated to enter the platoon. As mentioned in the message authentication phase, all existing platoon members will have the new platoon key pG shared with them by the PL. Hence, the primary objective is to allow the PL to distribute the new partial platoon key p to the existing members in its platoon. In this phase, the PL will send out an update request UpdateREQ and the partial platoon key p it generated to all current platoon members by attaching a timestamp and old platoon signature $SIG_{qG-7}$ to it. The platoon signature is similarly signed using the old partial platoon key q.

**Figure 4:**
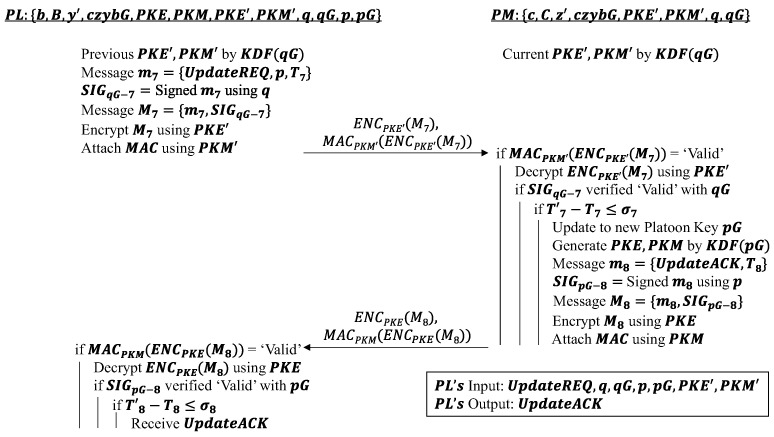
Platoon Key Update Phase of the SPMSA Scheme when a Vehicle Enters the Platoon.

Subsequently, the message is encrypted with a MAC attached to it. Once again, the previous set of platoon encryption and MAC keys $PKE^{\prime }$ and $PKM^{\prime }$ are used to carry these actions out. When the validity of the MAC, platoon signature and timestamp attached to the message sent are verified by the PM, the PM is able to derive the new platoon key pG and thereby generate a new set of platoon encryption and MAC keys PKE and PKM. A platoon key sends acknowledgment UpdateACK back to the PL to indicate the correct reception of the new platoon keys. This acknowledgment is accompanied by a timestamp and new platoon signature $SIG_{pG-8}$ before being encrypted and tagged with a MAC using the new keys PKE and PKM instead of the previous keys $PKE^{\prime }$ and $PKM^{\prime }$. The algorithm works as shown in Figure 4.

### 4.2. Platoon Communication Event

The platoon communication event refers to a scenario where successfully authenticated platoon members PMs intend to transmit payload information to other members during platooning. Message authentication is once again used, but this time its purpose is to conduct payload communication rather than transfer a platoon key. Let JV be the vehicle that has just joined the platoon and is the latest authenticated PM. Additionally, let an example scenario for this event be a PM informing JV to close the physical distance between them. The message exchange algorithm for this event is similar to that of the message authentication phase found in Section 4.1.3.

**Figure 5:**
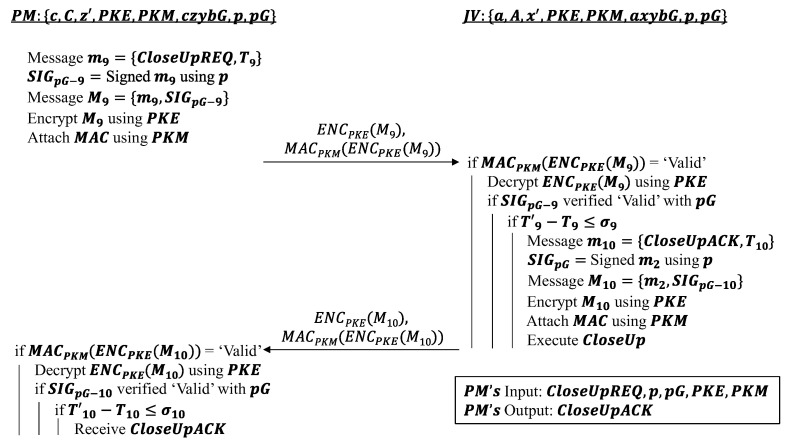
Platoon Communication Event of the SPMSA Scheme.

However, to protect the privacy of the vehicles in the platoon, the only information being sent over the communication channel of the platoon in this event is action requests and acknowledgements. In this instance, only CloseUpREQ and CloseUpACK messages are exchanged between the two vehicles. Each message is assigned with a timestamp and signed afterward using the partial platoon key p. The messages are then encrypted using the PKE key, and a MAC generated by the PKM key is attached to the resulting ciphertext. The receiving party of the request message, i.e., JV, then verifies the attached MAC and decrypts the ciphertext using the same set of PKE and PKM keys used by PM. If the platoon signature can be verified as valid using platoon key pG and the timestamp delta is less than the threshold $\sigma_{9}$, JV is able to acknowledge PM’s request and execute it. JV then replies to PM in an equivalent manner by attaching a timestamp and platoon signature to the message before encrypting it and tagging it with a MAC using the same PKE and PKM keys. At the end of the message exchange, PM receives an acknowledgement CloseUpACK informing it that JV is executing the CloseUp action. Figure 5 details the algorithm for this event.

### 4.3. Platoon Exit Event

A platoon exit occurs when a current platoon member informs its platoon leader that it wishes to leave the platoon. Once again, since it is an interaction between authenticated platoon members, the message comes from the leaving vehicle, and it should be confirmed that the message has not been tampered with by a malicious platoon member. Hence, only message authentication is used throughout the two phases of this event.

#### 4.3.1. Exit Request Phase

The aim of this phase is to allow a vehicle to leave the platoon without compromising the privacy of the platoon afterwards. For simplicity’s sake, it is assumed that the leaving vehicle is JV, which holds the same set of keys and maintains information after the platoon entry and communication events. The *JV* is denoted as LV to indicate it is a leaving vehicle. Once again, the algorithm for this phase is similar to that in Section 4.1.3 as it ultimately is the inverse operation of the message authentication phase. The contents of the message sent by LV contains a platoon leaving request LeaveREQ and a hashed 2-tuple of the current platoon key pG and LV’s hashed identity $H\left(ID_{A}\right)$ i.e., $H\left(pG,H\left(ID_{A}\right)\right)$. The composition of the message is the same as that of Section 4.2, where a timestamp and platoon signature are attached to the message before *PKE* and PKM keys are used to encrypt the message and tag it with a MAC.

On the reception of the encrypted message, the PL first checks the validity of the MAC, platoon signature and timestamp. If they are all valid, the PL then verifies if LV’s double identity is in the platoon members list PMList, i.e., if LV is an authenticated member of the platoon. If LV’s record is in the PMList, its entry is removed from the list and a platoon exit acknowledgment LeaveACK is sent back to the LV. Similar to the first message sent by LV in this phase, the same procedure is used by PL to prepare the message for transmission to the LV. At the same time, a partial platoon key r is generated by PL so that it can update its current platoon key pG to the latest key rG. This updated partial platoon key is then shared only with the other platoon members PMs that will be staying in the platoon.

**Figure 6:**
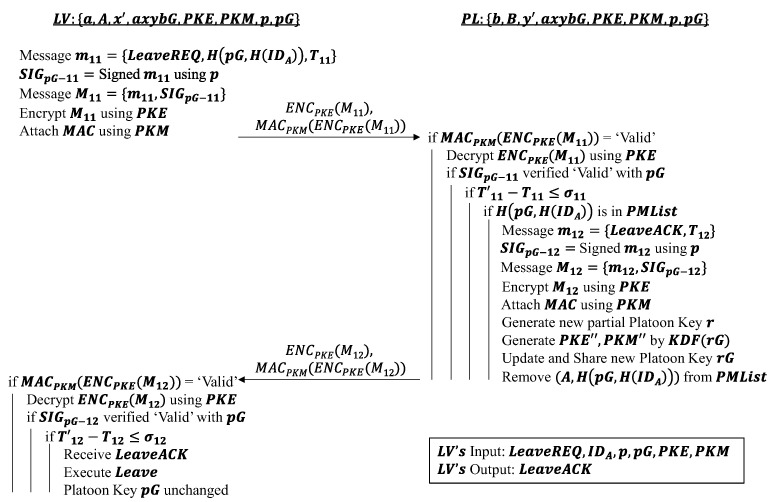
Exit Request Phase of the SPMSA Scheme.

Upon receipt of LeaveACK, the LV can leave the platoon as it has been deemed safe to exit. This is because the LV does not retain any significant information pertaining to the platoon and its members. For example, to ensure group forward secrecy, the platoon key rG being used by the platoon after LV’s exit is different from the one that LV still possesses, i.e., pG. The only information that LV retains with regards to the platoon is the PL’s long-term public key B. The algorithm for this phase can be found in Figure 6.

#### 4.3.2. Platoon Key Update Phase (Exit)

**Figure 7:**
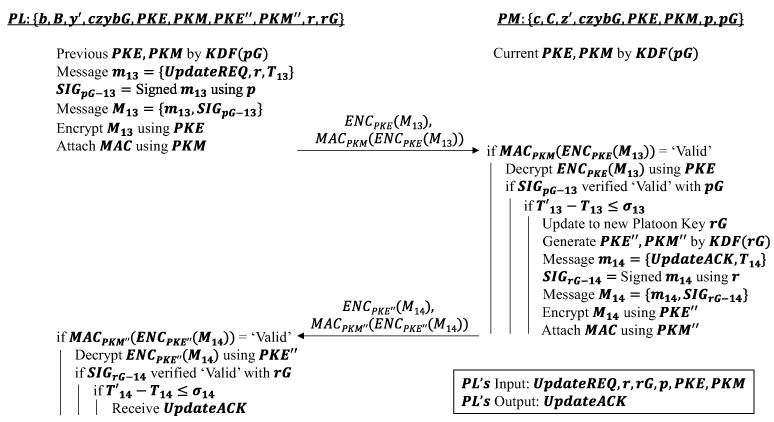
Platoon Key Update Phase of the SPMSA Scheme when a Vehicle Leaves the Platoon.

This phase is initiated when a vehicle is allowed to exit the platoon, and its primary goal is to allow the PL to distribute a new platoon key r to the PMs that remain in the platoon. Its operation is similar to that in the platoon key update phase in Section 4.1.4. The differences merely lie in the keys being used. From the exit request phase in Section 4.3.2, it is clear that the new platoon key to be used in the platoon is now rG instead of pG. Hence, the partial platoon key r needs to be shared with all PMs so that they can derive the new platoon key rG and preserve the privacy of the platoon from outgoing vehicle LV. The old platoon signature $SIG_{pG}$ is attached to the update request and new partial platoon key r before being sent over by using the old platoon encryption and MAC keys PKE and PKM. The usual verification of the received message is performed by the PM before it can safely generate the new set of platoon keys: rG, $PKE^{\prime \prime }$, $PKM^{\prime \prime }$. The new platoon signature $SIG_{rG}$ and timestamp are attached to the update acknowledgment UpdateACK and encapsulated as an encrypted message using the new $PKE^{\prime \prime }$ key. The new MAC key $PKM^{\prime \prime }$ is duly used to tag the encrypted message where the PL is able to verify its validity. The algorithm can be found in Figure 7 and concludes the SPMSA scheme.

## 5. Security Evaluation

### 5.1. Formal Proof of Security by Random Oracle Model

We first evaluate the SPMSA scheme formally by proving its semantic security under the CK adversary with random oracle model. With the CK adversary model, a probabilistic polynomial–time adversary 𝒜 can eavesdrop, modify and inject information into the message exchange process by interacting with the participants involved. In the random oracle model [23], there exists a random oracle that models cryptographic hash functions as ideally random functions. With this model, all participants can interact with one another including 𝒜. The queries covered in Section 3.2 that detail the various actions 𝒜 can take are assumed to be sent to this random oracle for execution [22].

#### 5.1.1. Formal Proof of Platoon Entry Event

Ultimately, the goal of adversary 𝒜 is to determine the real platoon key pG from a random number that occurs in the test query. It requires 𝒜 to break the semantic security of the SPMSA for the entry event. To evaluate whether the SPMSA can withstand 𝒜’s attempt, it is run through a series of games outlined by the random oracle model. In this section, we omit the initialization phase as it involves the preparation of the keying materials for the system rather than the running of the SPMSA. Meanwhile, the platoon key update phase will be verified in Section 5.2.1. Thus, the proof will only cover the identity and message authentication phases of the platoon entry event. Let $Pr\left[S_{i}\right]$ be the probability that 𝒜 succeeds in predicting the results of the test query for Game i. Let the joining vehicle and platoon leader be denoted by JV and PL. $q_{h}$, $q_{s}$, $q_{e}$ and q represent the number of hash, send, execute and total random oracle queries sent by 𝒜, respectively, while H denotes the hash space size such that $H=2^{Hash\;Length\;\left(in\;Bits\right)}$. As mentioned in Section 3.2, the entry event of the SPMSA offers semantic security under the CK adversary with random oracle model if the advantage for 𝒜 winning all the games is $Adv_{Entry}\left(𝒜\right)\leq \varepsilon$ for any sufficiently small value ε>0.

**Lemma** **1** (Difference Lemma). *Let* $E_{1}$*,* $E_{2}$*,* F *denote events in a certain probability distribution where* F *is known as the failure event. The two events* $E_{1}$ *and* $E_{2}$ *will execute in a similar manner as long as the failure event* F *does not happen, i.e.,* $E_{1}\wedge \neg F\Leftrightarrow E_{2}\wedge \neg F$*. As both* $Pr\left[E_{1}\right]$ *and* $Pr\left[E_{2}\right]$ *are between 0 and* Pr[F]*. The subsequent difference between the probabilities of the two events is* $\left|Pr\left[E_{1}\right]-Pr\left[E_{2}\right]\right|\leq Pr\left[F\right]$*.*

**Game 0:** This game is the initial attacking game set out in Section 3.2. It is a real attack by 𝒜 in the semantic security framework under a random model. Hence, the advantage to 𝒜 is:

(1) $$Adv_{Entry}\left(𝒜\right)=\left|2Pr\left[S_{0}\right]-1\right|$$

**Game 1:** 𝒜 launches a passive attack on both parties in the authentication agreement in this game. 𝒜 sends an Execute(JV, PL) query to acquire the information exchanged between both parties, which includes $\left\{A,B,A^{\prime },B^{\prime },SIG_{A-3},SIG_{B-4},\;ENC_{KE}\left(M_{5}\right),\;\;ENC_{KE}\left(M_{6}\right),\;MAC_{KM}\left(ENC_{KE}\left(M_{5}\right)\right),\;MAC_{KM}\left(ENC_{KE}\left(M_{6}\right)\right),T_{1},T_{2},T_{3},T_{4}\;\right\}$. 𝒜 is then unable to compute session key axybG and thus is unable to derive the symmetric decryption key KE. As such, 𝒜 cannot acquire partial platoon key p, which is encrypted in $ENC_{KE}\left(M_{6}\right)$. Thus, the probability that 𝒜 succeeds is:

(2) $$Pr\left[S_{1}\right]=Pr\left[S_{0}\right]$$

**Game 2:** This game follows Game 1, with 𝒜 now using send queries to initiate active attacks. The following events are omitted, however, as either event would cause the game to be over instantly:

Event $E_{1}$: The collision of the hash query outputs in different sessions. The birthday paradox states that $E_{1}$ happens with probability $\left|Pr\left[E_{1}\right]\right|\leq \frac{q_{h}^{2}}{2H}$.

Event $E_{2}$: The collision of the random numbers generated in different sessions. As the random numbers are only generated in send and execute queries, $\left|Pr\left[E_{2}\right]\right|\leq \frac{\;{\left(q_{s}+q_{e}\right)}^{2}}{2q}$.

As long as the ECDLP and ECCDH assumptions hold, 𝒜 does not have sufficient information to reconstruct the previous session key axybG to decrypt $ENC_{KE}\left(M_{2}\right)$ and obtain partial platoon key p. 𝒜 is also unable to establish a new ephemeral key x (or y) using a send query as it needs either JV’s or PL’s long-term private key to sign the message so that its identity can be verified by the other party. Therefore, according to the difference lemma, (3) is obtained as follows:

(3) $$\left|Pr\left[S_{2}\right]-Pr\left[S_{1}\right]\right|\leq \frac{q_{h}^{2}}{2H}+\frac{\;{\left(q_{s}+q_{e}\right)}^{2}}{2q}+q_{h}\cdot max\left\{Adv_{ECDLP}\left(𝒜\right),\;Adv_{ECCDH}\left(𝒜\right)\right\}$$

**Game 3:** Game 3 involves running Game 2 while 𝒜 then tries to guess the hash values KDF(axybG)=KE||KM and $H\left(ID_{A}\right)$ without querying the random oracle. If the guess is correct, the game is over. Thus, the resulting polynomial is:

(4) $$\left|Pr\left[S_{3}\right]-Pr\left[S_{2}\right]\right|\leq \frac{q_{s}^{2}}{2H}$$

**Game 4:** This game continues from Game 3 but with the consideration of semantic security. 𝒜 obtains any two of {a, b,x,y} by making ESReveal and corrupt queries to the random oracle. However, according to the CK adversary model, 𝒜 is unable to obtain both the long-term and ephemeral private keys of the same vehicle at the same time (e.g., acquiring JV’s *a* and x). As a result, 𝒜 is unable to recompute the session key axybG and subsequently obtain platoon key pG because it needs both private keys of the same vehicle. The only way it can do so is to solve the ECDLP and find a or x from A and $A^{\prime }$, respectively, or to solve the ECCDH problem. Thus, since Game 4 is similar to Game 3,

(5) $$\mathrm{Pr}\left[S_{4}\right]=Pr\left[S_{3}\right]$$

Subsequently, 𝒜 initiates a test query where an unbiased coin c is flipped. Since the probability of such an event is $\frac{1}{2}$,

(6) $$Pr\left[S_{4}\right]=\frac{1}{2}$$

Combining all the advantages from Game 0 to Game 4 i.e., Equations (1) to (6) through back substitution, we can obtain (7):

(7) $$Adv_{Entry}\left(𝒜\right)\leq \frac{q_{h}^{2}}{H}+\frac{\;{\left(q_{s}+q_{e}\right)}^{2}}{q}+\frac{q_{s}^{2}}{H}+2q_{h}\cdot max\left\{Adv_{ECDLP}\left(𝒜\right),\;Adv_{ECCDH}\left(𝒜\right)\right\}$$

Since $Adv_{Entry}\left(𝒜\right)\leq \varepsilon$, where ε>0, the entry event of the SPMSA is safe under the CK adversary with random oracle model.

#### 5.1.2. Formal Proof of Platoon Exit Event

For the formal proof of platoon exit, the platoon key update phase will also be verified in Section 5.2.1. Thus, we only evaluate Section 4.3.1 of the SPMSA, i.e., the exit request phase. We evaluate a scenario where JV in the platoon is now looking to exit the platoon. Following the notation in Figure 6, JV is synonymous with LV. It is assumed that no vehicle has joined or left the platoon since *LV*’s entry into the platoon. In other words, the platoon key that is established throughout all platoon members is pG.

A goal of adversary 𝒜 is to determine the current platoon key pG from a random number that occurs in the test query. Since a vehicle leaving the platoon causes the platoon key to be updated, 𝒜 must intercept the LeaveREQ from LV to prevent it from reaching PL. It ensures that the platoon key 𝒜 obtained will remain valid for use in the platoon and not be outdated. The notations used for the formal proof of the platoon entry phase are reused here. There is no change to the lemma difference or the games conducted. Similarly, we can say that the exit phase of our scheme offers semantic security under the CK adversary with the random oracle model if the advantage for 𝒜 winning all of the games is $Adv_{Exit}\left(𝒜\right)\leq \varepsilon$ for any sufficiently small ε>0.

**Game 0:** This is the initial attacking game set out in Section 3.2, which outlines a real attack by 𝒜 in the semantic security framework under a random model. Hence, the advantage of 𝒜 is the same as that of (1).

**Game 1:** A passive attack on both parties is first launched by 𝒜 in this game. 𝒜 sends an Execute(LV, PL) query to steal the information exchanged between the parties, which includes $\left\{ENC_{PKE}\left(M_{11}\right),ENC_{PKE}\left(M_{12}\right),\;MAC_{PKM}\left(ENC_{PKE}\left(M_{11}\right)\right),\;\;MAC_{PKM}\left(ENC_{PKE}\left(M_{12}\right)\right)\right\}$. Since 𝒜 does not hold platoon key pG, it is unable to decrypt the information it has stolen as it cannot generate the necessary platoon encryption and MAC keys PKE and PKM. Hence, 𝒜 cannot obtain partial platoon key r, and the probability that 𝒜 succeeds is found in (2).

**Game 2:** Once again, this game follows Game 1 with 𝒜 using send queries thereafter to initiate active attacks. Similarly, the following events are omitted:

Event $E_{1}$: The collision of the hash query outputs in different sessions. The birthday paradox states that $E_{1}$ happens with probability $\left|Pr\left[E_{1}\right]\right|\leq \frac{q_{h}^{2}}{2H}$

Event $E_{2}$: The collision of the random numbers generated in different sessions. As the random numbers are only generated in send and execute queries, $\left|Pr\left[E_{2}\right]\right|\leq \frac{\;{\left(q_{s}+q_{e}\right)}^{2}}{2q}$

Only if the two events above occur will 𝒜 have a plausible amount of information to potentially forge a legitimate message to intercept the communication between LV and PL. Therefore, according to the Difference Lemma, (8) is obtained as follows:

(8) $$\left|Pr\left[S_{2}\right]-Pr\left[S_{1}\right]\right|\leq \frac{q_{h}^{2}}{2H}+\frac{\;{\left(q_{s}+q_{e}\right)}^{2}}{2q}$$

**Game 3:** After the conclusion of Game 2, 𝒜 tries to guess the hash values KDF(pG)=PKE||PKM and $H\left(pG,H\left(ID_{A}\right)\right)$ without querying the random oracle. Recall that the platoon encryption and MAC keys PKE and PKM are generated from KDF(pG). If the guess is correct, the game is over, and the resulting polynomial is (4).

**Game 4:** Following Game 3, semantic security is taken into consideration. 𝒜 obtains any two of $\left\{a,\;b,x^{\prime },y^{\prime }\right\}$ by making ESReveal and corrupt queries to the random oracle. However, obtaining any of them will not enable 𝒜 to procure platoon key pG. This is because the queries only uncover the components of session key axybG. As shown in Figure 6, this key is not involved in this phase. Hence, with no additional advantage for 𝒜 to obtain platoon key *pG*, Game 4 is no different from Game 3 which can be seen in (5).

𝒜 then initiates a test query in which an unbiased coin c is flipped and the probability of such an event is $\frac{1}{2}$, as shown in (6).

Finally, we combine the advantages from Game 0 to Game 4, i.e., Equations (1), (2), (4), (5), (6) and (8) through back substitution to get the advantage of 𝒜, as seen in (9).

(9) $$Adv_{Exit}\left(𝒜\right)\leq \frac{q_{h}^{2}}{H}+\frac{\;{\left(q_{s}+q_{e}\right)}^{2}}{q}+\frac{q_{s}^{2}}{H}$$

Since $Adv_{Exit}\left(𝒜\right)\leq \varepsilon$, where ε>0, the platoon exit event of the SPMSA is also safe under the CK adversary with random oracle model.

### 5.2. Formal Verification of Security Functionality by CryptoVerif

In this section, we verify the security functionality of the platoon key update phases of the SPMSA that we covered in Section 4.1.4 and Section 4.3.2. Although these phases occur in two different events, the algorithms are the same, with the only difference the names of the keys. Hence, verifying the security of one of the platoon key update phases could verify the security of another.

First, we briefly review CryptoVerif. CryptoVerif is an automatic protocol verifier on security that is sound in the computational model. It can verify secrecy and correspondences such as authentication. It provides formal verifications as a sequence of games, similar to the CK adversary model that we used to prove the other parts of the SPMSA. However, instead of being manually implemented, CryptoVerif can be automatically executed via a programming model. The generated verifications are valid for any number of sessions of the protocol. Hence, it can provide an upper bound on the probability of the success of an adversary against the protocol as a function of the likelihood of breaking each cryptographic primitive and of the number of sessions it takes to do so [25].

**Figure 8:**
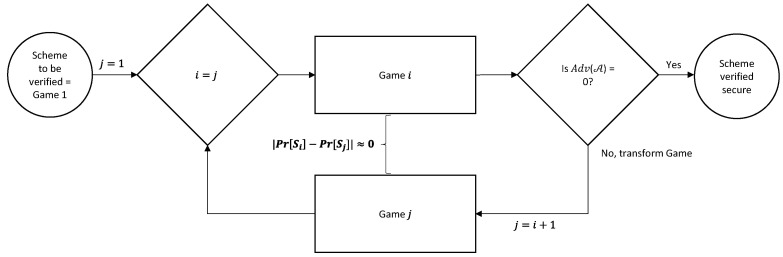
Security Verification by the Game-Hopping Process of CryptoVerif.

The input script for CryptoVerif to run contains the cryptographic assumptions and properties to verify. CryptoVerif uses the technique of game hopping where the first game models the actual protocol that we wrote in the input script to verify. From the second game onwards, CryptoVerif applies syntactic transformations on the game until the game satisfies the security properties realized. Note that an adversary is unable to distinguish one game from another after transformation as the difference of probability between consecutive games is negligible, i.e., |Pr[Si]−Pr[Sj]|≈0, where j=i+1. Consequently, the advantage of the adversary for the final game is Adv(𝒜)=0. Figure 8 shows the game-hopping procedure of the CryptoVerif. After CryptoVerif finishes execution, it will output the sequence of games that occurred, a brief explanation of the transformations that took place between the games and finally, the upper bound of probability of an adversary being successful against the protocol [26]. For our formal verification results, we show the first and last games and the upper bound probability of the adversary of breaking the security properties of the SPMSA.

#### 5.2.1. Formal Verification of Platoon Key Update Phases

As mentioned beforehand, the platoon key update phases for entry and exit events have the same algorithm, with the name of the keys being the only difference. The actors involved in both phases are the same, i.e., a platoon leader and its members, and the messages exchanged are of the same structure. Specifically, the messages being exchanged exist in the structure of an encrypted message $ENC_{PKE}\left(M\right),\;$and a MAC $MAC_{PKM}\left(ENC_{PKE}\left(M\right)\right)$, where M is the plaintext encapsulated message, while PKE and PKM are the platoon encryption and MAC keys, respectively. Since the algorithm encrypts the plaintext message before attaching a MAC of the encrypted message, we can therefore deem it an Encrypt-then-MAC cryptographic scheme.

CryptoVerif has a library of predefined cryptographic primitives that can be used to model the SPMSA scheme. For the platoon key update phases of the SPMSA scheme, the core principle is Encrypt-then-MAC. We use the following primitives that have already been specified in CryptoVerif’s library [26]:

- Expand IND_CPA_sym_enc(key, cleartext, ciphertext, enc, dec, injbot, Z, Penc).
  This primitive defines an indistinguishable under a chosen plaintext attack (IND-CPA) probabilistic symmetric encryption scheme. In other words, given the encryption of two messages of the same length, an adversary has a negligible probability of telling the two encryptions apart. We denote this probability as Penc.
- Expand SUF_CMA_det_mac(mkey, macinput, macres, mac, check, Pmac).
  This primitive defines a strongly unforgeable under chosen message attacks (SUF-CMA) deterministic MAC. This means that for an adversary that is given access to the MAC and verification oracles, it has a negligible probability of forging a MAC. This probability is denoted as Pmac.

We use the oracle front-end of CryptoVerif, which is more suitable in our case because its syntax of games resembles manual cryptographic verification better. This falls in line with the previous proofs of the SPMSA scheme in Section 5.1 that were performed manually. We adopt the input scripts written by the author in [25] to verify two security properties of the platoon key update phases of the SPMSA scheme: that the encryption of plaintext message is indistinguishable (IND-CPA) and that the integrity of the ciphertext generated by the encryption is hard to break (INT_CTXT). By verifying these two properties, we can safely say that the partial platoon key, update request and timestamp are transferred securely to the PMs with the SPMSA.

For the IND-CPA property verification, two oracles called L and R are required. CryptoVerif uses equivalences to transform the processes that call the L oracles into processes that call the R oracles. If the oracles on the two sides return different results, the event is deemed unreachable, and CryptoVerif declares that the two sides, i.e., the encryption of messages, are indistinguishable.

**Figure 9:**
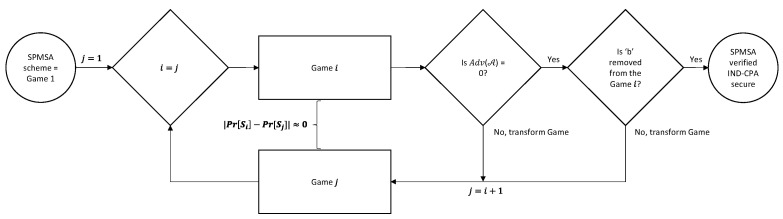
Game-Hopping to Verify IND-CPA Property of Platoon Key Update Phases.

To verify the IND-CPA property in a discernible manner, a query secret Boolean *b* is used where if *b* = 1, then message = *m*1, while if *b* = 0, message = *m*2. After a specific game transformation, if *b* has no influence on which message is encrypted, then we can confirm the IND-CPA property [26]. Figure 9 shows the process of this verification.

**Figure 10:**
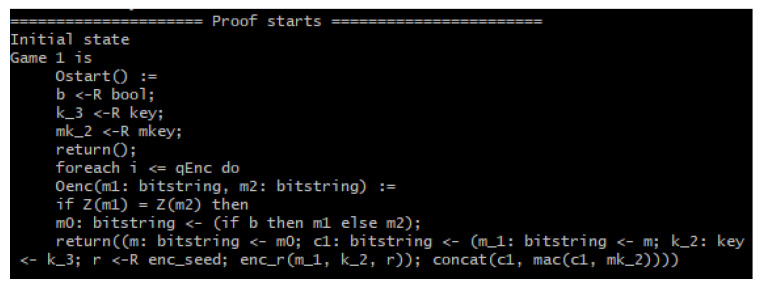
First Game of Verifying the IND-CPA Property of Platoon Key Update Phases.

**Figure 11:**
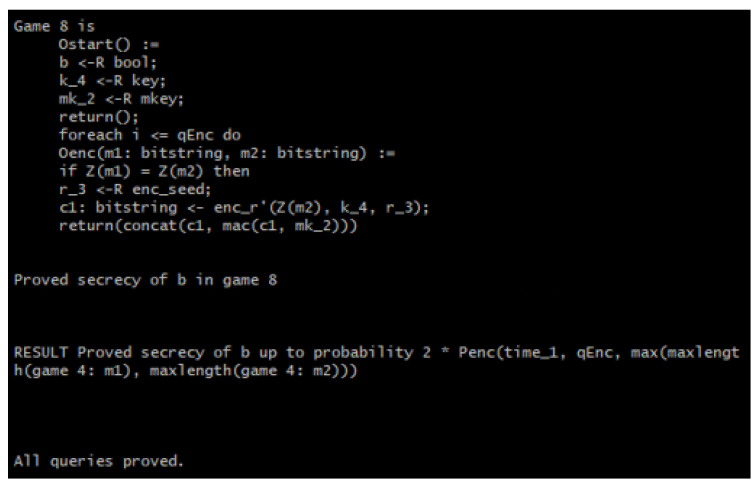
Final Output of IND-CPA Verification of Platoon Key Update Phases.

Figure 10 shows the initial game of the verification, while Figure 11 shows it takes eight games (seven game transformations) for the query secret *b* to not be used in the games anymore because the line of code containing *b* is missing and the game goes straight into encrypting the message. This is because CryptoVerif merges the two “if” branches of the test “*m*0: bitstring <- (if *b* then *m*1 else *m*2);” as the same code to be executed in either branch. In short, *m*1 and *m*2 are indistinguishable because the two use the same code. Finally, the RESULT header shows the upper bound probability of the adversary to be successful in telling the encryption of messages apart. This upper bound is shown to be double that of Penc, which is the probability of breaking the IND-CPA property of the underlying encryption scheme, as previously discussed.

**Figure 12:**
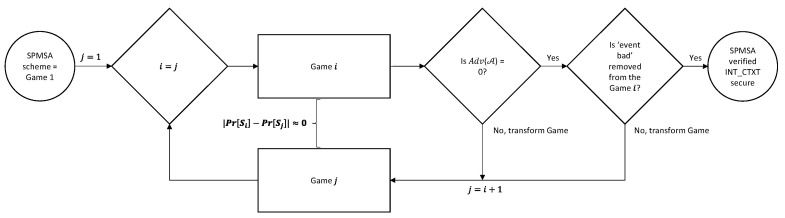
Game-Hopping to Verify INT_CTXT Property of Platoon Key Update Phases.

For the INT_CTXT property verification, encryption and decryption test oracles are required. A query event “bad” is used to show whether the adversary has successfully broken the INT_CTXT property. If event bad occurs, the adversary has managed to produce a ciphertext that decrypted successfully and has not been produced by the encryption oracles. Hence, the verification is only successful when event bad does not happen, i.e., when the occurrence of event bad is false [26]. Figure 12 shows the process of the INT_CTXT property verification.

**Figure 13:**
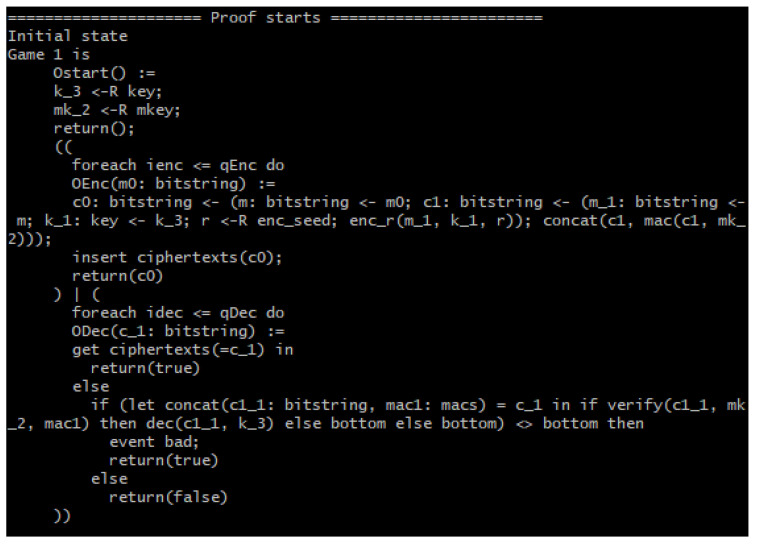
First Game of Verifying the INT_CTXT Property of Platoon Key Update Phases.

**Figure 14:**
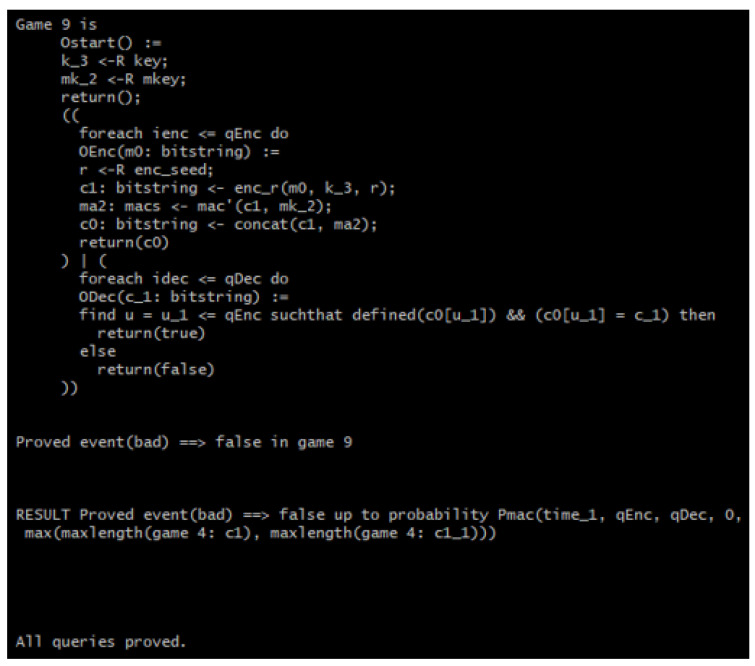
Output of INT_CTXT Verification of Platoon Key Update Phases.

Figure 13 portrays the initial game of the INT_CTXT verification where event bad can be seen in the fifth and last line. The final result of the INT_CTXT security verification in Figure 14 shows that nine games (eight game transformations) are required for event bad to no longer occur in the game. Hence, CryptoVerif has verified that the adversary will not be able to forge a ciphertext that can be decrypted successfully and has not been produced by the encryption oracles. Finally, the RESULT header shows the upper bound probability that the adversary will be successful in breaking the ciphertext integrity to be equivalent to Pmac, which is the probability of breaking the SUF-CMA property of the MAC.

To conclude, through the use of a computational verifier tool CryptoVerif, we showed that the platoon key update phases of the SPMSA resist an adversary 𝒜 distinguishing between encrypted messages. It is also resistant to 𝒜 forging ciphertexts that can be decrypted to obtain the original plaintext message, which in our case crucially includes the partial platoon key p for the entry event and r for the exit event.

#### 5.2.2. Formal Verification of Security Functionality for Communication Event

In fact, by nature, the communication event is a much more simplified version of the key update phases. They both have a pair of request and acknowledgment messages exchanged using Encrypt-then-MAC. However, the platoon communication event involves purely payload communication. In comparison, the key update phases require an additional partial platoon key to be transmitted over the channel to generate the platoon key. Transferring these additional data does not make the algorithms more complex; rather, it introduces additional potential vulnerability.

Thus, since we have verified that the key update phases are secure by CryptoVerif, we can then deduce that the algorithm of the platoon communication event that has fewer potential data vulnerabilities is secure as a result. To conclude, the platoon communication event resists an adversary 𝒜 distinguishing between its encrypted messages as well as 𝒜 forging ciphertexts of valid plaintext messages. To reiterate, these messages only include platoon requests/acknowledgments and the accompanying timestamps.

### 5.3. Security Analysis

In this section, a qualitative analysis of the security properties and the abilities against some of the typical malicious attacks of the SPMSA scheme is presented.

Mutual authentication: Mutual authentication can be achieved by both identity and message authentication as discussed in Section 4.1.2 and Section 4.1.3. Digital signatures are used to ensure the identity of the vehicle, while MACs are used to confirm the message’s origin and integrity.

As stated in Section 3.2, the Canetti–Krawczyk (CK) adversary model was used against the SPMSA to test it for any vulnerabilities. Participants, partners and the adversary are the parties involved based on the model. The adversary represents a Sybil vehicle that can make queries to disrupt and obtain information to authorize itself as a legitimate platoon member. The adversary’s main goal is to obtain a valid and working platoon key by tethering the communication between any pair of partners, including JV/LV, PL and RSU. With the help of the CK threat model, the SPMSA is secure against Sybil attackers.

Key agreement: The session key axybG and platoon key pG can be computed by both JV and PL after the mutual authentication. As long as the long-term private key is inaccessible and the ECDLP and the ECCDH assumptions hold, an attacker cannot compute the session key.

Perfect forward secrecy: The previously established session key axybG will still be secure even if the long-term private keys a and b are compromised. This is due to an attacker’s inability to obtain the previous ephemeral private keys x or y, which have expired and have been erased from the OBU’s memory.

Group backward secrecy: Whenever a vehicle joins a platoon, even if the platoon is one it has joined before, it cannot compute or possess the previously used platoon key qG.

Group forward secrecy: Whenever a vehicle leaves a platoon, it is unable to compute or possess the new platoon key rG.

Ability against replay attacks: A replay attack is launched so that 𝒜 can spoof a legitimate vehicle by sending previous data to the vehicles. Adding short-term keys generated by random numbers and timestamps can ensure the freshness of messages.

Ability against man-in-the-middle (MitM) Attacks: With a MitM attack, 𝒜 tries to establish connections with vehicles individually to make them mistakenly believe that they are connected to each other. In the entry event of the SPMSA, where JV and PL try to set up a session key and new platoon key, we mentioned in Game 4 of Section 5.1.1 that an attacker cannot acquire both the long-term and the ephemeral private keys of the same vehicle at the same time. Hence, even if the messages are intercepted and modified by 𝒜, it cannot have the generator point G forge a signature that will be validated by JV and PL. The other phases of the scheme involve authenticated communication between platoon members PMs, so 𝒜 can only establish a connection with vehicles individually if it has the platoon key. Otherwise, a MitM attack will fail.

Ability against distributed denial-of-service (DDoS) attacks: A DDoS attack is an attack in which multiple malicious attackers overwhelm a single server/node to deny other nodes access to it. Identity authentication should be able to detect and disregard these malicious vehicles in time before the platoon leader is overwhelmed.

Ability against Sybil attacks: We discussed in Section 4 that Sybil vehicles can be detected by the SPMSA with a combination of identity and message authentications. This detection can be achieved by invalidating timestamps, digital signatures, MACs and hashed vehicle identities that accompany transmitted messages. When detected, the message and session pertaining to that Sybil vehicle are discarded afterwards, and the vehicle is denied entry into the platoon.

## 6. Performance Evaluation

The performance of the SPMSA was evaluated by estimating computation and communication overheads as well as simulation experiments to determine the average elapsed time. We primarily contrasted the performance of the SPMSA with that of PASAD in [9]. We deem it a comparable and appropriate scheme to use for platooning purposes, in large part due to its group membership phase.

Since the platoon entry event is where the major performance issues lie, only Section 4.1 of the SPMSA is considered for the entirety of the performance evaluation. For a fair comparison of the two schemes, we only apply PASAD when a vehicle joins a new RSU group. This is because this event can plausibly resemble a platoon entry event where a vehicle tries to join a platoon. Hence, only Algorithms 4–7 of PASAD are considered. Since this instance of PASAD only involves the initial authentication of vehicles for entry into the new RSU group, we also exclude the platoon key update phase in Section 4.1.4 of the SPMSA from this point onwards.

For reference, the PASAD scheme in [9] is executed through seven algorithms. We shall provide a brief description of each algorithm as follows:

Algorithm 1: System initialization that is conducted by the Center of Authority (CA) to generate the common parameters for the TRSUs and RSUs to register the vehicles.

Algorithm 2: Generation of the first private key for a vehicle by a TRSU.

Algorithm 3: Generation of a signature by a vehicle to prove its unique existence.

Algorithm 4: RSU ensures no double-registrations of vehicles entering its group by verifying the signature provided by the entering vehicle.

Algorithm 5: Group initialization by TRSU or RSU to generate the local group parameters as well as the vehicles’ secondary private key.

Algorithm 6: A vehicle that has joined a group generates a signature for issuing event-reporting messages.

Algorithm 7: Verification of signatures by the vehicles which have received a specific event from another vehicle within the group.

### 6.1. Computational Overheads

The various cryptographic operations that comprise the SPMSA and PASAD are estimated to find the computational delay of these schemes. We adopt the evaluation method in [27] using the following parameters in our experiments including an Intel Core i3 2.4-GHz processor with MIRACL and Crypto++ libraries. Table 1 summarizes the execution times of the cryptographic operations.

To calculate the computational delay of the two schemes, we only consider time-consuming operations that are not involved in the initialization stages. Hence, Section 4.1.1 of the SPMSA and Algorithm 5 of PASAD were excluded. Low computational modular arithmetic operations such as addition and subtraction were also excluded. The computational delay for the SPMSA scheme is calculated in (10):

(10) $$T_{SPMSA}=4T_{ECIES}+4T_{ECDSA-S}+4T_{ECDSA-V}+6T_{Mul}+2T_{Hash}=72.558\;\mathrm{ms}$$

We assume a best-case scenario where there are no invalid signatures i.e., no Sybil nodes. The computational costs for Algorithms 4, 6 and 7 of PASAD are as follows:

(11) $$T_{PASAD-Alg4}=2T_{Mul}+4T_{Pair}$$

(12) $$T_{PASAD-Alg6}=8T_{Exp}+7T_{Mul}+T_{Pair}+T_{Hash}$$

(13) $$T_{PASAD-Alg7-Best}=9nT_{Exp}+\left(9n+2\right)T_{Mul}+\left(2n+4\right)T_{Pair}$$

To create a similar environment to compare with the SPMSA, we assume a minimalist VANET system for PASAD where two vehicles communicate in isolation as in the SPMSA. Hence, n=1, and the total computation overhead of PASAD is

(14) $$T_{PASAD}=T_{PASAD-Alg4}+T_{PASAD-Alg6}+T_{PASAD-Alg7-Best}=323.1189\;\mathrm{ms}$$

Comparing (10) and (14) shows that the SPMSA has an estimated lower time complexity than that of PASAD.

### 6.2. Communication Overheads

We estimate the communication delay of the two schemes by calculating the total sum of transmission and propagation delays of each. The transmission delay is the amount of time to transmit the packets of data onto the transmission medium. It can be determined by L/R, where L is the size of a data packet in bits and R is the data transmission rate in bits per second (bps). We can assume that the data rates of V2V and V2I communication, i.e., DSRC and 5G, to be 6 and 50 Mbps, respectively.

To determine the transmission delay, we first approximate the amount of memory required to run the two schemes. The keys and hashes used in the SPMSA scheme require 256 bits of data each as the secp256r1 and SHA256 protocols have been used as the initial parameters of the elliptic curve and hash function, respectively. In addition, the MACs attached to the encrypted messages also take up 256 bits each. Meanwhile, plaintext messages and timestamps require 32 bits each, while each ECDSA signature uses up 512 bits. Lastly, an additional parity bit is needed for each key and signature. It culminates in a data size of 5642 bits being transmitted by the SPMSA scheme. Meanwhile, assuming that PASAD also uses the ECIES for its symmetric encryption, it requires an estimated 5324 bits to be transmitted [9].

On the other hand, propagation delay is the amount of time for the packets of data to reach the destination over the physical medium. The formula for the propagation delay is thus D/S, where D is the physical distance between the two vehicles and S is the propagation speed of the communication link. On average, we assume that a single V2V link covers a distance of 50 m while a V2I link covers about 200 m. In both cases, the communication is wireless, so a common propagation speed of the speed of light ($3\times 10^{8}\;m/s$) can be assumed. For each scheme, the number of transmissions that occur using DRSC and 5G technology are summed up to determine their respective propagation delays. We ignore queuing and processing delays as they are dependent on several factors that we cannot predict well. The communication delays of the two schemes as well as that of another relevant scheme by Santhosh et al. [8] can be found in Table 2.

It can be seen that the propagation delay makes a tiny contribution to the overall communication delay for all schemes. Instead, the communication overhead is predominantly determined by the transmission delay. Both PASAD and the SPMSA performed considerably better than the scheme by Santhosh et al.

Focusing only on PASAD and the SPMSA, they portrayed comparable communication delays despite the SPMSA requiring a couple hundred more bits to be transmitted than PASAD. However, since their respective communication delays are relatively insignificant when compared with the computational overheads for both schemes, the computational overhead will be the dominant factor in the execution times of the schemes.

### 6.3. Performance Comparison by Simulations

In this section, we evaluate the performance of the SPMSA using simulations. First, we observe its performance under some unknown attacks and compare it with that of PASAD. The simulation is built on MATLAB software. A known attack is an attack that should have been picked up by the SPMSA or by PASAD. In contrast, an unknown attack is one that was not analyzed or claimed previously. Intuitively, numerous unknown attacks could be launched at any time. They could break the execution of a protocol, and if they did so, they would likely break it at different points of execution. The probability that they will do either is difficult to predict with full certainty. Thus, we model these two processes as independent random processes with uniform probability. In turn, the objective of this simulation is to predict the negative effects to the performance of the system of unknown attacks.

In the simulation, one million attacks were launched for each specified ratio of the unknown attacks to the total attacks, with ratio ranging from 0 to 0.8 in increments of 0.1. An unknown attack has a uniform probability of breaking the scheme at a random step in the execution of the protocol. Note that the execution time of a protocol is defined as the sum of the computational overhead and communication delay that has been calculated beforehand in Section 6.1 and Section 6.2. If a protocol can resist an attack, we consider the scheme successful. However, when an unknown attack breaks the scheme, only the execution time up till the point the scheme stops running is recorded. Subsequently, it is not deemed a successful run. For a given ratio of unknown attack:

(15) $$Average\;Execution\;Time=\frac{Total\;Execution\;Time\;After\;1\;Million\;Attacks}{Number\;of\;Successful\;Runs\;of\;Scheme}$$

**Figure 15:**
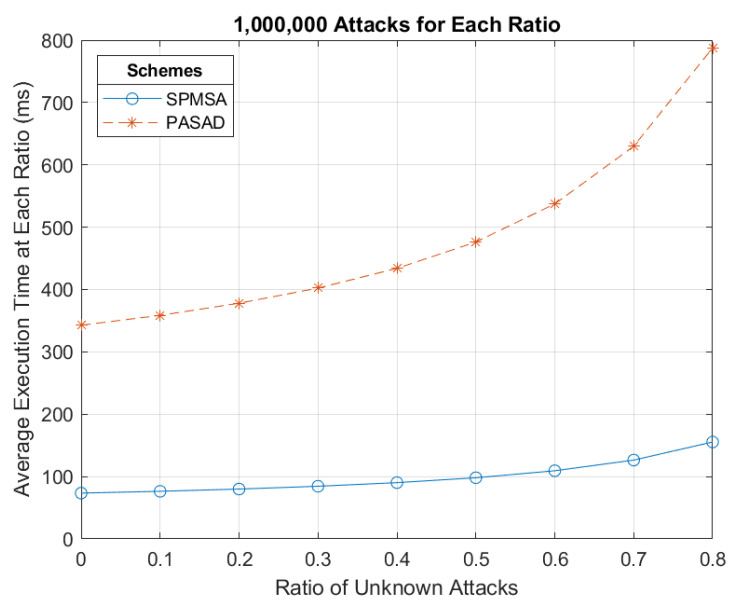
Performance of the Two Schemes against Unknown Attacks.

The average execution times of the SPMSA and PASAD at each ratio of unknown attacks is plotted in Figure 15. When the ratio of unknown attacks is 0, it represents both the execution time as well as the communication reconnection time of the schemes in the event of disturbances such as channel interference that was discussed in Section 1. There is an exponential increase in the execution/reconnection times of both schemes that is to be expected when there are more unknown attacks obstructing the schemes from completion. More importantly, our SPMSA is less time-consuming than PASAD regardless of the ratio of the unknown attacks that appear, peaking at 155.4 ms while achieving a similar major goal of preventing Sybil attacks.

We conducted a second simulation experiment to evaluate the performance of the SPMSA when Sybil nodes were present in the VANETs. We compared the performance with regards to the time it takes to authenticate all honest vehicles with that of PASAD. Let n represent the number of vehicles intending to join the platoon, while x represents the number of Sybil vehicles among these n vehicles. We assume an average-case scenario of PASAD this time around. Hence, the equation in (13) becomes (16), and the total computation cost of PASAD is seen in (17):

(16) $$\begin{array}{ll} T_{PASAD-Alg7-Avg} & =9nT_{Exp}+\left(3n\log x+x\log\left(\frac{n}{x}\right)+11n-x+\frac{n\left(n+1\right)}{2}\right)T_{Mul}\\ & +\left(2x\log\left(\frac{n}{x}\right)+4x+n\left(n+1\right)\right)T_{Pair} \end{array}$$

(17) $$T_{PASAD-Avg}=T_{PASAD-Alg4}+T_{PASAD-Alg6}+T_{PASAD-Alg7-Avg}$$

Since the SPMSA discards the message and session of a detected Sybil vehicle, only the time taken to reach such an occurrence was recorded by the scheme as the authentication time of a Sybil vehicle. In contrast, in the case of the authentication of an honest vehicle, the time taken for a full run of the SPMSA was recorded. For both schemes, we assume that at least one honest node is present in the VANET such that n−x≥1. In this simulation, we set n to be 9 throughout and vary the number of Sybil vehicles x from 1 to 8. Thus, there will be eight independent runs of the simulation to outline the authentication time cost of each scheme when the number of Sybil vehicles varies. For example, in the first run, there is x=1 Sybil vehicle among the n=9 vehicles that would like to join the platoon. The total time taken to authenticate all 9 vehicles is cumulatively summed up and denoted by $T_{Auth}$. To calculate the average authentication time $\bar{T_{Auth}}$, the cumulative authentication time is divided by the number of honest vehicles, as seen in (18). This process will then be repeated for x=2, 3,…,8 for both schemes.

(18) $$\bar{T_{Auth}}=\frac{T_{Auth}}{n-x}$$

**Figure 16:**
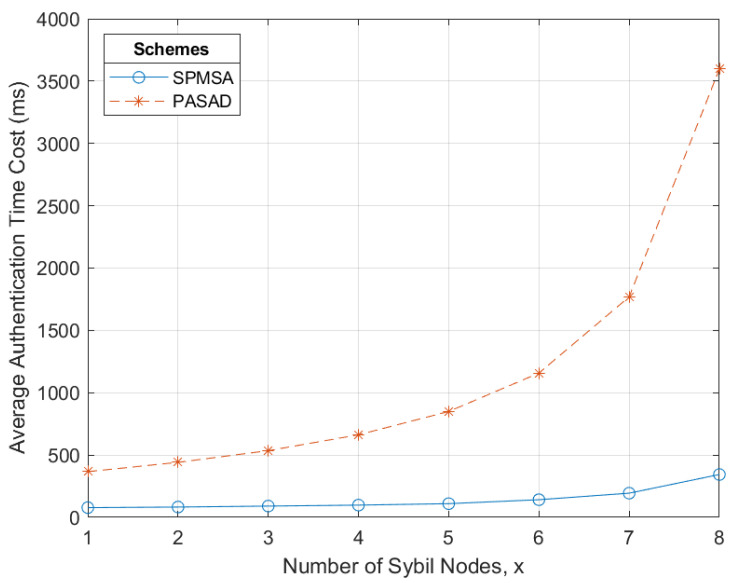
Performance of the Two Schemes in the Presence of Sybil Nodes.

The average authentication times for the two schemes for a varying number of Sybil vehicles is plotted in Figure 16. As more Sybil vehicles are added, the difference in performance of the two schemes in terms of authentication time cost grows greater. The performance achieved by PASAD is not a surprise as its computational delay increases considerably when there are many invalid signatures, as mentioned in Section 2.3. More notably, the number of Sybil vehicles in the VANET is not a factor in determining which scheme is faster in authenticating honest vehicles as our scheme has been shown to be consistently faster. At its peak, PASAD takes roughly 3.6 s. In comparison, the SPMSA only consumes approximately 0.34 s.

## 7. Conclusions

In this paper, we have proposed a secure management scheme for platoon access that is resistant to Sybil attacks using elliptic curves. The SPMSA can achieve both identity and message authentication between a platoon leader and a vehicle intending to join the platoon and maintain message authentication throughout the vehicle’s tenure in the platoon. The security functionality of the proposed SPMSA was then proven in the CK adversarial model with the random oracle model as well as with the CryptoVerif protocol verifier. We also conducted a qualitative analysis of the scheme’s security to show its security features including perfect forward secrecy and both group forward and backward secrecy. Finally, we evaluated the performance of the proposed scheme with numerical analysis and simulation experiments to show its time efficiency in the face of unknown attacks and the minimal resource costs when Sybil vehicles are present. Future work is expected to explore the authentication of a new platoon leader when the existing leader intends to leave the platoon. As evidenced by the proposed scheme, the platoon leader carries important information that needs to be handed over to the right vehicle in a secure manner.

## Figures and Tables

**Table 1:** Execution Time of Cryptographic Operations.

Notation	Description	Execution Time (ms)
Pair	Bilinear Pairing Operation	23.625
Exp	Exponentiation Operation	3.3421
Mul	Scalar Multiplication	1.258
Hash	SHA256 Hash Function	0.005
ECIES	Operation of ECIES	4.35
ECDSA−S	Signing Operation of ECDSA	3.01
ECDSA−V	Verifying Operation of ECDSA	8.89

**Table 2:** Comparison of the Communication Delays of Schemes.

	PASAD	SPMSA	Santhosh [8]
Transmitted Data (bits)	5324 (DSRC)	5354 (DSRC) 288 (5G)	8192 (DSRC)
Transmission Delay (ms)	0.88733	0.89809	1.36533
Number of Transmissions	3 V2I (DSRC)	6 V2V (DSRC) 2 V2I (5G)	6 V2V (DSRC) 2 V2I (DSRC)
Propagation Delay (ms)	0.00200	0.00233	0.00233
Total Communication Delay (ms)	0.88933	0.90042	1.36767

## Data Availability

The data presented in this study are available in the article.

## References

[1] Boeira F., Barcellos M.P., de Freitas E.P., Vinel A., Asplund M. On the Impact of Sybil Attacks in Cooperative Driving Scenarios. Proceedings of the 2017 IFIP Networking Conference and Workshops.

[2] Boeira F., Barcellos M.P., de Freitas E.P., Vinel A., Asplund M. Effects of colluding Sybil nodes in message falsification attacks for vehicular platooning. Proceedings of the 2017 IEEE Vehicular Networking Conference (VNC).

[3] Solyom S., Coelingh E. (2013). Performance Limitations in Vehicle Platoon Control. IEEE Intell. Transp. Syst. Mag..

[4] Vahidi A., Eskandarian A. (2003). Research advances in intelligent collision avoidance and adaptive cruise control. IEEE Trans. Intell. Transp. Syst..

[5] Samara G., Al-Raba’nah Y. (2015). Security Issues in Vehicular Ad Hoc Networks (VANET): A survey. Int. J. Sci. Appl. Res..

[6] Sarker A., Qiu C., Shen H. (2020). Connectivity Maintenance for Next-Generation Decentralized Vehicle Platoon Networks. IEEE ACM Trans. Netw..

[7] Rabieh K., Mahmoud M.M., Guo T.N., Younis M. Cross-Layer Scheme for Detecting Large-scale Colluding Sybil attack in VANETs. Proceedings of the 2015 IEEE International Conference on Communications (ICC).

[8] Santhosh J., Sankaran S. Defending against Sybil Attacks in Vehicular Platoons. Proceedings of the 2019 IEEE International Conference on Advanced Networks and Telecommunications Systems (ANTS).

[9] Parham M., Pouyan A.A. (2020). An Effective Privacy-Aware Sybil Attack Detection Scheme for Secure Communication in Vehicular Ad Hoc Network. Wirel. Pers. Commun..

[10] Soni M., Jain A. Secure Communication and Implementation Technique for Sybil Attack in Vehicular Ad-Hoc Networks. Proceedings of the 2018 Second International Conference on Computing Methodologies and Communication (ICCMC).

[11] Kushwah R., Kulshreshtha A., Singh K., Sharma S. ECDSA for Data Origin Authentication and Vehicle Security in VANET. Proceedings of the 2019 Twelfth International Conference on Contemporary Computing (IC3).

[12] Bochem A., Leiding B., Hogrefe D. Unchained identities: Putting a price on sybil nodes in mobile ad hoc networks. Proceedings of the International Conference on Security and Privacy in Communication Systems.

[13] Bochem A., Leiding B. (2021). Rechained: Sybil-resistant distributed identities for the Internet of Things and mobile ad hoc networks. Sensors.

[14] Liu X., Luo B., Abdo A., Abu-Ghazaleh N., Zhu Q. Securing Connected Vehicle Applications with an Efficient Dual Cyber-Physical Blockchain Framework. Proceedings of the 2021 IEEE Intelligent Vehicles Symposium (IV).

[15] Didouh A., Lopez A.B., Hillali Y.E., Rivenq A., Faruque M.A.A. Eve, You Shall Not Get Access! A Cyber-Physical Blockchain Architecture for Electronic Toll Collection Security. Proceedings of the 2020 IEEE 23rd International Conference on Intelligent Transportation Systems (ITSC).

[16] Gu P., Khatoun R., Begriche Y., Serhrouchni A. Support Vector Machine (SVM) Based Sybil Attack Detection in Vehicular Networks. Proceedings of the 2017 IEEE Wireless Communications and Networking Conference (WCNC).

[17] Quevedo C.H.O.O., Quevedo A.M.B.C., Campos G.A., Gomes R.L., Celestino J., Serhrouchni A. An Intelligent Mechanism for Sybil Attacks Detection in VANETs. Proceedings of the ICC 2020—2020 IEEE International Conference on Communications (ICC).

[18] Mohanti S., Soltani N., Sankhe K., Jaisinghani D., Di Felice M., Chowdhury K. AirID: Injecting a custom RF fingerprint for enhanced UAV identification using deep learning. Proceedings of the GLOBECOM 2020—2020 IEEE Global Communications Conference.

[19] Reus-Muns G., Jaisinghani D., Sankhe K., Chowdhury K.R. Trust in 5G Open RANs through Machine Learning: RF Fingerprinting on the POWDER PAWR Platform. Proceedings of the GLOBECOM 2020—2020 IEEE Global Communications Conference.

[20] Comert C., Kulhandjian M., Gul O.M., Touazi A., Ellement C., Kantarci B., D’Amours C. (2022). Analysis of Augmentation Methods for RF Fingerprinting under Impaired Channels. Proceedings of the 2022 ACM Workshop on Wireless Security and Machine Learning (WiseML’22).

[21] Canetti R., Krawczyk H., Pfitzmann B. (2000). Analysis of Key-Exchange Schemes and Their Use for Building Secure Channels. Proceedings of the International Conference on the Theory & Application of Cryptographic Techniques.

[22] Chen W.-C., Huang Y.-T., Wang S.-D. (2021). Provable Secure Group Key Establishment Scheme for Fog Computing. IEEE Access..

[23] Bellare M., Pointcheval D., Rogaway P., Preneel B. (2000). Authenticated Key Exchange Secure against Dictionary Attacks. Proceedings of the International Conference on the Theory and Applications of Cryptographic Techniques.

[24] Martínez V.G., Encinas L.H., Dios A.Q. (2015). Security and Practical Considerations When Implementing the Elliptic Curve Integrated Encryption Scheme. Cryptologia.

[25] Blanchet B. (2017). CryptoVerif: A Computationally-Sound Security Protocol Verifier.

[26] Blanchet B., Cadé D. (2021). CryptoVerif Computationally Sound, Automatic Cryptographic Protocol Verifier User Manual.

[27] Pan J., Cui J., Wei L., Xu Y., Zhong H. (2019). Secure data sharing scheme for VANETs based on edge computing. J. Wirel. Com. Netw..

